# Morphological and biological characterization of a light‐colored mutant in the multicolored Asian lady beetle, *Harmonia axyridis*

**DOI:** 10.1002/ece3.4379

**Published:** 2018-10-03

**Authors:** Yuan‐Xing Sun, Ya‐Nan Hao, Yu Yan, Yi Zhang, Yi Feng, Tong‐Xian Liu

**Affiliations:** ^1^ Key Laboratory of Integrated Pest Management on Crops in Northwestern Loess Plateau Ministry of Agriculture State Key Laboratory of Crop Stress Biology for Arid Areas Northwest A&F University Yangling China; ^2^ Biocontrol Engineering Laboratory of Crop Diseases and Pests of Gansu Province College of Plant Protection Gansu Agricultural University Lanzhou China

**Keywords:** body color, fitness, *Harmonia axyridis*, melanin, mutant, pleiotropy

## Abstract

1. Insect cuticle color formed with melanin pigments has numerous types of mutants which usually cause pleiotropic effects. Melanism has been widely studied, but mutants with light‐colored phenotype as well as the consequent fitness changes have rarely been reported.
2. Here, in the laboratory strain of *Harmonia axyridis*, we found a novel mutant *gr* and confirmed that the mutation was inherited in a simple Mendelian autosomal recessive manner. This mutant (HAM) continuously displayed a light‐colored pigmentation versus dark blackish in the wild phenotype (HAW). L‐DOPA and dopamine are melanin precursors, and less L‐DOPA was present in the cuticle of larval and adult HAM mutants compared to HAW wild type, but more dopamine was detected in the larval cuticle of HAM (*p *≤* *0.0235). For the orange background of elytra, the composition as well as total concentration of carotenoids was different between HAM and HAW, which resulted in significantly lower saturation value but significantly higher hue value in HAM than in HAW (*p *<* *0.0001).
3. Extensive fitness changes were detected in HAM. (a) HAM larvae had similar predation capacity and preimaginal development time as HAW, but the newly emerged adults were much smaller (*p *<* *0.0001). (b) Both fecundity and egg hatch rate in cross ♀(HAM) × ♂(HAM) were significantly lower than those in ♀(HAW) × ♂(HAW) (*p *≤* *0.0087), but were not different with those in ♀(HAW) × ♂(HAM). (c) HAM had weaker resistance to desiccation and ultraviolet irradiation compared to HAW (*p *≤* *0.0115).
4. These results indicated that the novel light‐colored mutant (HAM) was highly correlated with fitness changes, and it would be a perfect model to study molecular mechanisms of melanism and how a gene results in pleiotropic effects.

## 1 INTRODUCTION

Pigmentation is vital for animals because it can protect them from the harmful effects of UV irradiation, reduce the risk of being preyed upon, facilitate sexual selection, and so on (Protas & Patel, 2008). Melanin is the predominant class of insect pigments which make the cuticle display dark coloration (Sugumaran, 2002; Wittkopp & Beldade, 2009). In addition, this substance has been shown to be involved in a wide range of vital adaptive functions, such as camouflage, photoprotection, sexual signaling, thermoregulation, protection against reactive oxygen species, strengthening insect cuticles, and immune defense (reviewed by Krams et al. (2016)). Melanogenesis is a complex multistep production of melanins via hydroxylation, oxidation, and polymerization of the oxidized metabolites (Singh, Malhotra, Pandey, & Pandey, 2013). To be specific, melanin biosynthesis in insect cuticle initially starts with tyrosine being converted to dopa by tyrosine hydroxylase (TH), and the dopa is directly used to produce dopa‐melanin pigments or further converted to dopamine with the action of dopa decarboxylase (DDC) and used to produce dopamine‐melanin pigments (Wright, 1987; Zhang et al., 2017). During these processes, genetic changes usually resulted in body color changes, in which melanism has been reported to be the most common type of mutant (True, 2003).

In insects, melanism mutations have been widely studied and shown to be tightly related to fitness changes. For example, late emergence was detected in the *nigra* homozygotes of the scalloped hazel moth, *Odontoptera bidentata* (Cook & Jacobs, 1983), but faster development was found in the melanic phenotype of the peppered moth, *Biston betularia* (Bruce & Cyril, 1999). In addition, of the melanism phenotype, increased fecundity in the army worm, *Mythimna separata* (Jiang, Luo, & Zhang, 2007) and the forest tent caterpillar, *Malacosoma disstria* (Lorimer, 1979), but opposite results were found in the cotton bollworm, *Helicoverpa armigera* (Ma, Chen, Wang, & Li, 2008) and the polyphenic butterfly, *Bicyclus anynana* (Bear, Simons, Westerman, & Monteiro, 2010).

There are few reports related to light‐colored mutants, with the exception of several albinism mutants that totally lack melanin pigmentation (Sugahara, Tanaka, Jouraku, & Shiotsuki, 2017). Albino mutants have been observed in several insect species including locusts (Faure, 1932), the cave‐dwelling plant hopper, *Oliarus polyphemus* (Bilandžija, Ćetković, & Jeffery, 2012), the silkworm, *Bombyx mori* (Fujii et al., 2013; Liu et al., 2010; Ohnuma, 2001), and the fruit fly, *Drosophila melanogaster* (Biessmann, 1985; Wittkopp, True, & Carroll, 2002). The albino phenotypes of the migratory locust, *Locusta migratoria* and the desert locust, *Schistocerca gregaria* were both shown to be controlled by a simple Mendelian unit (Hasegawa & Tanaka, 1994; Hunter‐Jones, 1957). Albinism generally causes deleterious effects on fitness (Bilandžija et al., 2012), and several albinism mutants of *B. mori* were reported to be lethal, for example, the mutant albino (*al*) immediately died after the first ecdysis (Fujii et al., 2013). Overall, studies related to fitness changes of light‐colored mutants were quite limited, but these studies would be very important to elucidate the function of melanin pigments in insects and to reveal the pleiotropy effects of a gene involved in melanin production.

The multicolored Asian lady beetle, *Harmonia axyridis* (Pallas), a well‐known polyphagous predator, is wildly distributed around the world (Koch, 2003). Under natural conditions, the larva displays gray to dark blackish coloration in the first instars or with orange stripes covering the dorsal and lateral areas in later instars (Koch, 2003; Sasaji, 1977). In our laboratory strain, we accidentally discovered several neonate larvae with goldenrod coloration (*gr*) hatching from the egg clusters of one female, and the abnormal phenotypes occurred throughout all development stages as compared with the wild type (HAW). In addition, the light‐colored phenotypes were confirmed to be expressed in stable inheritance after continuous rearing and inbreeding for several generations. Thus, we supposed that this abnormal phenotype might be a novel mutant (indicated as HAM) deficient in melanin pigment production and would be an applicable model to study what fitness changes occurred in a melanin deficiency mutation. For *H. axyridis* adults, form *succinea* (the background of elytra is yellow or yellow with black spots [Tan, 1946]), the yellow‐orange to red part on elytra is due to deposition of carotenoids, another class of pigments; while the dark spots are formed with melanin (Bezzerides, McGraw, Parker, & Husseini, 2007). Carotenoids, which must be acquired from diets, are important antioxidants and immunostimulants and play multiple roles in vision, diapause, and photoperiodism (Olson & Owens, 1998; Svensson & Wong, 2011). Thus, HAM is also an ideal model to study the functional correlation of carotenoids‐ and melanin‐based pigmentation.

In this study, a series of experiments were conducted to elucidate the features of this mutant. First, the typical phenotype and pigment deposition in cuticle were imaged with light microscopy; and the contents of two important melanin precursors (L‐DOPA and dopamine) were measured to identify the physiological basis of cuticle color changes. Coloration of the elytra and deposition features of carotenoids were measured to reveal the changes of melanin‐ and carotenoid‐based pigmentation. Then, classical crossing experiments between HAM and HAW were conducted to characterize whether the mutation is (a) recessive, co‐dominant, or dominant; (b) autosomal or sex‐linked; and (c) controlled by one or more loci. Finally, the life history traits and adaptive capabilities of this mutant were determined to measure the extent of fitness changes.

## 2 MATERIALS AND METHODS

### 2.1 Insects rearing and strain establishment

Two pairs of *H. axyridis* adults were collected from a wheat field in Yangling, Shaanxi, China (34°17′00″N, 108°03′42″E) in April 2013 and were continuously reared in an insectary under 25 ± 1°C, 65% RH and 14:10 h L:D conditions. The lady beetles were fed with *Myzus persicae* (Sulzer) on pepper leaves. In November 2014, several neonate larvae with abnormal goldenrod coloration (*gr*) were accidentally discovered from the egg clusters of one couple of *H. axyridis* adults. The *gr* larvae were collected and raised with *M. persicae*, and individuals in the third generation were used in all subsequent experiments.

### 2.2 The phenotype and physiological changes

#### 2.2.1 Cuticle

Each developmental stage of the lady beetles (adults were collected at 48 hr postemergence) was killed with the vapor of alcohol, and their typical phenotypes (elytra and hind wings were dissected from the adult body) were photographed using a stereomicroscope (SMZ1500, Nikon Corporation, Tokyo, Japan) under the same conditions (light intensity, 5,600K).

To examine the content of two melanin precursors, L‐DOPA and dopamine, 6‐mg intact cuticle of the fourth instar larvae or lyophilized body of newly emerged adults (without elytra) were placed in a 1.5‐ml centrifuge tube, and crushed by grinding into homogenate in 1‐ml extraction solution (0.05 M HCl in 50% ethyl alcohol). The homogenate was then centrifuged at 20,000 *g* for 10 min at 4°C, and the supernatant was analyzed by an LTQ XL linear ion trap mass spectrometer (Agilent 1100, Thermo Scientific, Waltham, MA, USA) (Zhang et al., 2016). Larval samples of each phenotype were measured with eight replicates, and, due to limited samples of HAM, five replicates were measured for female newly emerged adults (in HAW, no significant difference was detected between female and male adults).

#### 2.2.2 Elytra

The left elytron of newly emerged adults was photographed with a digital camera (EOS M5, Canon Corporation, Tokyo, Japan), and the camera parameters were set in Manual (shutter speed: 1/200; ISO: 200; white balance: color temp, 5,600K). Photographs were imported into Adobe Photoshop (Adobe Systems, San Jose, CA, USA) following the methods described by Bezzerides et al. (2007) to obtain HSB (hue, saturation, and brightness) values of the orange background and brightness value of the dark spots (males were excluded for analysis due to only a few spots presented on the elytra).

After that, the elytra were weighed using a micro‐balance (Sartorius MSA 3.6P‐000‐DM, Got‐tingen, Germany) and then were individually added to 1 ml of chloroform to remove carotenoids as described by Bezzerides et al. (2007). The extracts were centrifuged to dryness in a vacuum concentrator (ScanSpeed 40, Scanvac, Ballerup, Denmark) and then were re‐dissolved in 500 μl alcohol. After being centrifuged at 13,000 *g* for 5 min, the supernatant was measured at 475 nm with an absorbance reader (TECAN infinite M200, Mannedorf, Switzerland). Each phenotype had 15 replicates, and a series of different concentrations of β‐carotene were used to draw the standard curve (Blount et al., 2012; Winters, Stevens, Mitchell, Blomberg, & Blount, 2014).

### 2.3 Determining the inheritance pattern of *gr*

To determine whether the mutant *gr* is a recessive allele, a series of single pair crosses of *gr* and the wild type (*wt*) were performed. The couples were separately kept in clean plastic Petri dishes (9 cm in diameter) and supplied with sufficient *M. persicae* (approximately 200 mg) on pepper leaves. The eggs in each Petri dish were checked once a day and incubated in a new plastic Petri dish (3 cm in diameter). The average number of eggs oviposited by one female, and the number of newly hatched larvae (identifying the phenotype) was recorded for 10 days. In total, 12 pairs were used for each cross.

To further assess whether *gr* lies at a single locus or at multiple loci, a series of single pair inbreeding crosses were performed between the F_1_ hybrid individuals. Meanwhile, backcrosses were performed between the F_1_ heterozygotes and the presumptive homozygotes *grgr*. The number of eggs oviposited was checked and recorded following the same procedure as described above.

### 2.4 Fitness changes of the *gr* mutation

#### 2.4.1 Predation capability and development

Predation capability of HAM and HAW was compared using *M. persicae* as the target prey on pepper leaf disck. In total, 60, 100, and 100 adult aphids were supplied for each second, third, and fourth instar larva, respectively. Before the experiment, the aphids were kept at 4 ± 1°C for 1 hr to minimize nymph production. After a 20‐min recovery time, one larva (starved for 12 hr) was introduced into the Petri dish and maintained for 24 hr. The number of aphids consumed was recorded. Fifteen larvae were measured for each instar.

In addition, the preimaginal development time of HAW and HAM feeding on *M. persicae* were compared under the same conditions (25 ± 1°C, 65% RH and 14:10 h L:D). Newly hatched larvae were individually kept in plastic Petri dishes (3 cm in diameter) and supplied with sufficient aphids until pupation. Time used to finish the preimaginal development was recorded, and newly emerged adults were weighted using a micro‐balance (Mettler Toledo AL204, Shah Alam Selangor, Malaysia). In total, 20 larvae were recorded for each phenotype.

#### 2.4.2 Reproductive capability

Reciprocal crosses between HAM and HAW were conducted in plastic Petri dishes. Each couple was supplied with approximately 200 mg *M. persicae* as food for oviposition for 10 days. The average number of eggs deposited and larval hatch rate was recorded each day, and 12 couples were used for each crossing group.

#### 2.4.3 Desiccation and ultraviolet resistance

Weight loss was used as a proxy for desiccation resistance (Parkash, Rajpurohit, & Ramniwas, 2008). This experiment was conducted following the methods described by Chen et al. (2016). The fourth instar larvae (12–24 hr postecdysis) were weighed and then individually transferred to a centrifuge vial (50 ml) containing allochroic silica gel, and the treatment without desiccant was used as control. Thirty‐six hours later, the larvae were removed from the tubes and weighed again to calculate the weight loss. Twenty‐five individuals were measured for each group.

Percentage of survival was used to determine the ultraviolet resistance. Twenty first instar larvae (12–24 hr old) were transferred to a plastic Petri dish (3 cm in diameter) that was restricted with a piece of gauze on the outside surface with a rubber band. Four Petri dishes (two for each of HAM and HAW) were placed under UVA irradiation (365.0 nm, WFH‐204B, Shanghai, China) for 2 hr. After irradiation, the lady beetles were transferred to a clean plastic Petri dish (9 cm in diameter) and supplied with sufficient *M. persicae* on pepper leaves. Survival of the larvae was checked 24 hr later. Treatments that followed the same procedures but free of UVA‐irradiation were conducted as a control. Twenty‐five replicates were used for each treatment.

### 2.5 Statistical analysis

Analysis of the crossing experiments was based on the null hypothesis that the goldenrod cuticle coloration was inherited in a simple Mendelian autosomal recessive manner. A chi‐squared test was run for each cross to determine whether the progeny ratio of *wt* to *gr* differed significantly from the expected ratio (3:1 for the inbreeding crosses of F_1_ hybrids, and 1:1 for the backcross of F_1_ hybrids and recessive homozygotes). The reproductive parameters were analyzed with one‐way analysis of variance (ANOVA), and the data for desiccation and ultraviolet resistance were analyzed using two‐way ANOVA. Means were separated using Tukey's HSD test (*p *<* *0.05). The differences of other parameters between HAM and HAW were analyzed with independent sample *t* test (*p *<* *0.05). All data analyses were conducted with the SAS 9.1 software (SAS Institute 2010).

## 3 RESULTS

### 3.1 The phenotype and physiological changes

#### 3.1.1 Phenotype

**Figure 1:**
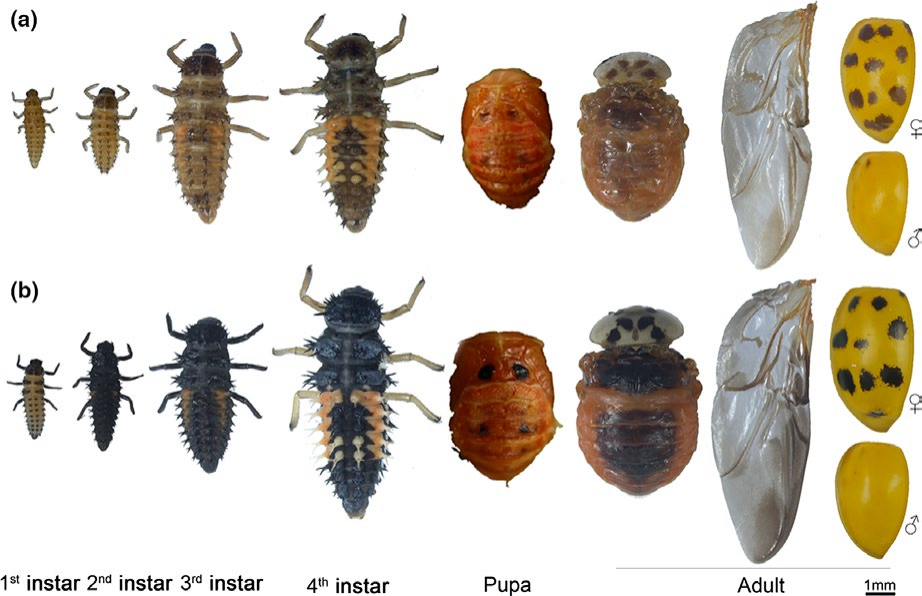
The typical morphology of different stages of *Hamonia axyridis* mutant type (HAM) and wild type (HAW). Photographs were the dorsal view and taken under the same conditions

The light‐colored phenotype was observed to be continuously expressed throughout all development stages (Figure 1). HAM displayed goldenrod coloration on larval cuticle at the areas which were gray to dark black in HAW. Dark spots on pupae were also lighter on HAM than on HAW. In adults, similar color change was found on hind wings, pronotum, dorsal abdomen, and spots on the elytra, and it was surprising to note that colors of the orange background area of the elytra were also different between HAM and HAW (Figure 1). Specifically, the brightness value of the dark spots on elytra was significantly higher (less dark) in HAM than in HAW (*df* = 28, *t *=* *18.29, *p *<* *0.0001) (Table 1). Of the orange background area, saturation value of HAM was significantly lower than that of HAW (*df* = 28, female: *t *=* *57.78, *p *<* *0.0001; male: *t *=* *148.50, *p *<* *0.0001), but the hue value was higher (*df* = 28, female: *t *=* *21.97, *p *=* *0.0022; male: *t *=* *34.89, *p *<* *0.0001). The brightness value was similar between the two phenotypes (*df* = 28, female: *t *=* *0.80, *p *=* *0.3801; male: *t *=* *0.20, *p *=* *0.6550) (Table 1).

**Table 1:** Brightness value of dark spots and HSB (hue, saturation, and brightness) values of orange background of the elytra of newly emerged adults

		Dark spots	Orange background		
		Brightness (100%)	Hue (°)	Saturation (100%)	Brightness (100%)
Female	HAM	42.53 ± 0.80*	48.20 ± 0.34*	89.00 ± 1.15	68.67 ± 0.50
	HAW	26.53 ± 0.95	46.93 ± 0.43	97.87 ± 0.37*	68.00 ± 0.55
Male	HAM		48.47 ± 0.33*	91.00 ± 0.82	69.47 ± 0.49
	HAW		47.27 ± 0.35	97.61 ± 0.46*	69.07 ± 0.74

*Note*

Values shown were mean ± *SE*, and asterisk indicates a significant difference between HAM and HAW (*p* < 0.05, *t* test).

#### 3.1.2 Physiological changes

**Figure 2:**
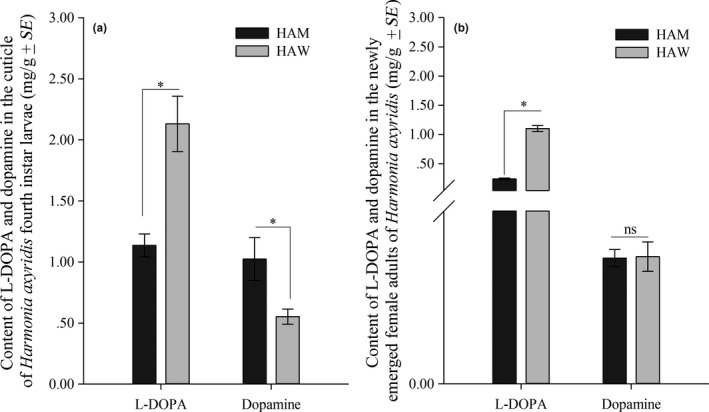
The concentration of L‐DOPA and dopamine in the cuticle of fourth instar larvae (a) and newly emerged female adults (b) of *Harmonia axyridis* mutant type (HAM) and wild type (HAW). Asterisk represents a significant difference between HAM and HAW (*p *<* *0.05, independent sample *t* test)

**Figure 3:**
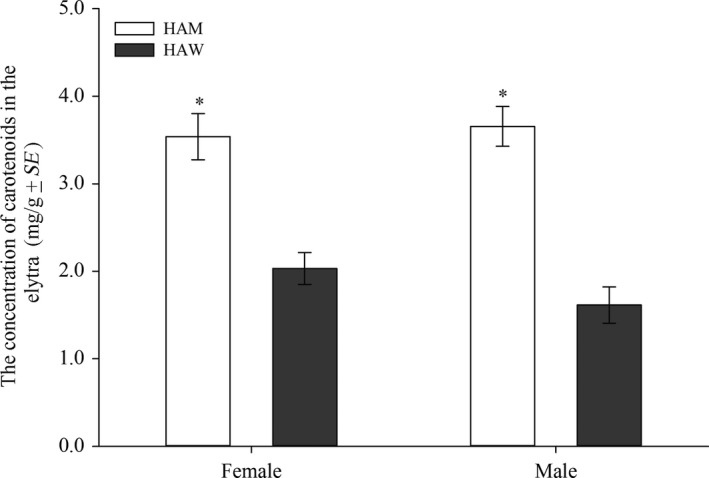
The concentration of carotenoids in the elytra of newly emerged adults of *Harmonia axyridis* mutant type (HAM) and wild type (HAW). For both female and male, asterisk represents a significant difference between HAM and HAW (*p *<* *0.05, independent sample *t* test)

In order to elucidate the physiological basis of the color changes (melanin‐based cuticle color and carotenoid‐based elytra color) of HAM, the contents of two melanin precursors (L‐DOPA and dopamine) and carotenoids were compared between HAM and HAW. Compared to HAW, the fourth instar larvae of HAM had significantly lower concentration of L‐DOPA (*df* = 14, *t *=* *−4.06, *p *=* *0.0012), but had significantly higher concentration of dopamine in their cuticles (*df* = 14, *t *=* *2.54, *p *=* *0.0235) (Figure 2a); the newly emerged female adults of HAM also had significantly lower concentration of L‐DOPA (*df* = 8, *t *=* *−16.30, *p *<* *0.0001), but no significant difference was detected for dopamine (*df* = 8, *t *=* *−0.08, *p *=* *0.9392) (Figure 2b). In elytra, the total concentration of carotenoids of female HAM (3.54 mg/g) was significantly higher than that of HAW (2.03 mg/g) (*df* = 28, *t *=* *22.07, *p *<* *0.0001), and similar differences were found in males (HAM: 3.65 mg/g, HAW: 1.62 mg/g) (*df* = 28, *t *=* *44.13, *p *<* *0.0001) (Figure 3). Moreover, through HPLC analysis, the composition of carotenoids was also different between HAM and HAW (Supporting Information Figure S1).

### 3.2 Determining the inheritance pattern of *gr*

The abnormal phenotype of HAM was expressed throughout development, and we hypothesized that this mutant was inherited in a simple Mendelian autosomal recessive manner. Based on this hypothesis, the progeny of *grgr* × *wtwt* would be 100% wild type, and the F_1_ heterozygotes should carry two alleles, one from each parent, resulting in 25% goldenrod phenotype in the F_2_ generation when sibs of the F_1_ offspring were mated. Additionally, when the F_1_ heterozygotes were mated with homozygous *grgr*, 50% of the offspring were expected to display goldenrod phenotype. Results from the crossing experiments were in accordance with theorized expectations and confirmed that the mutation *gr* is homozygous recessive at a single locus (Tables 2 and 3). Due to pleiotropic effects, this light‐colored mutation of *H. axyridis* (HAM) might express a wide range of fitness changes, including biological characteristics and desiccation and ultraviolet resistance.

**Table 2:** Frequencies of *wt* and *gr* offspring from the inbreeding crosses of the F1 heterozygotes of *wtwt* × *grgr*, with an expected ratio of 3:1 basing on the null hypothesis that the mutant was formed with two recessive alleles . The exceptional significant *p* values were highlighted in bold

Female parent	Male parent	Offspring phenotype		Chi‐square test	
		#WT	#GR	χ^2^	*p*
F1 *wtwt*♀ × *grgr*♂	F1 *wtwt*♀ × *grgr*♂	54	11	2.262	0.133
		120	37	0.172	0.678
		63	22	0.035	0.851
		109	133	0.235	0.628
		35	11	0.029	0.865
		161	43	1.673	0.196
		72	23	0.032	0.859
		120	35	0.484	0.487
F1 *grgr*♀ × *wtwt*♂	F1 *grgr*♀ × *wtwt*♂	138	44	0.066	0.797
		158	74	5.885	**0.015**
		172	62	0.279	0.597
		24	6	0.400	0.527
		286	120	4.496	**0.034**
		129	38	0.449	0.503
		121	20	8.797	**0.003**
		117	41	0.076	0.783
		65	18	0.486	0.486
		39	14	0.057	0.812
F1 *grgr*♀ × *wtwt*♂	F1 *wtwt*♀ × *grgr*♂	225	74	0.010	0.920
		153	44	0.746	0.388
		227	76	0.001	0.974
		18	7	0.120	0.729
		76	22	0.340	0.560
		115	45	0.833	0.361
		25	5	1.111	0.292
		58	23	0.498	0.480
		51	8	4.119	**0.042**
F1 *wtwt*♀ × *grgr*♂	F1 *grgr*♀ × *wtwt*♂	152	45	0.489	0.484
		46	22	1.961	0.161
		115	36	0.108	0.742
		68	21	0.094	0.760
		84	27	0.027	0.869
		116	31	1.200	0.273
		241	69	1.243	0.265
		179	49	1.497	0.221
		205	54	2.380	0.123
		177	43	3.491	0.062
		67	36	5.440	**0.020**

**Table 3:** Frequencies of *wt* and *gr* offspring from the backcrosses of the F1 heterozygote of *wtwt* × *grgr* and homozygote *grgr*, with an expected ratio of 1:1 basing on the null hypothesis that the mutant was formed with two recessive alleles . The exceptional significant *p* values were highlighted in bold

Female parent	Male parent	Offspring phenotype		Chi‐square test	
		#WT	#GR	χ^2^	*p*
F1 *wtwt*♀ × *grgr*♂	P0 *grgr*	51	39	1.600	0.206
		17	16	0.030	0.862
		114	107	0.222	0.638
		48	36	1.714	0.190
		149	146	0.031	0.861
		73	75	0.027	0.869
P0 *grgr*	F1 *wtwt*♀ × *grgr*♂	37	24	2.770	0.096
		37	40	0.117	0.732
		44	37	0.605	0.437
		77	91	1.167	0.280
		31	29	0.067	0.796
		93	100	0.254	0.614
		86	70	1.641	0.200
		32	25	0.860	0.354
F1 *grgr*♀ × *wtwt*♂	P0 *grgr*	11	7	0.889	0.346
		20	25	0.556	0.456
		25	14	3.103	0.078
		30	21	1.558	0.208
		53	57	0.145	0.703
		161	170	0.245	0.621
		49	30	4.570	**0.033**
P0 *grgr*	F1 *grgr*♀ × *wtwt*♂	23	22	0.022	0.881
		89	68	2.809	0.094
		43	42	0.012	0.914
		39	33	0.500	0.480
		15	5	5.000	**0.025**
		36	29	0.754	0.385
		125	118	0.202	0.653

### 3.3 Fitness changes of the *gr* mutation

#### 3.3.1 Larval predation capacity and development

The second, third, and fourth instar larvae of HAM, respectively, consumed 9.7, 28.8, and 65.1 *M. persicae* within 24 hr, which were slightly less than those eaten by individuals of HAW (12.9, 30.3, and 67.2, respectively), and a significant difference was detected in the second instar larvae (*df* = 28, 2nd instar: *t *=* *3.08, *p *=* *0.0046; 3rd instar: *t *=* *0.63, *p *=* *0.5357; 4th instar: *t *=* *0.38, *p *=* *0.7072) (Table 4). The development time of larva‐adult of HAM and HAW were similar, approximately 12 days (*df* = 38, *t *=* *0.00, *p *=* *1.000). However, the body weights of newly emerged adults of HAM were significantly lower than those of HAW (*df* = 38, female: *t *=* *4.16, *p *=* *0.0002; male: *t *=* *5.34, *p *<* *0.0001) (Table 4).

**Table 4:** The predation capacity and development of *Harmonia axyridis* mutant type (HAM) and wild type (HAW) on *Myzus persicae*

	Number of *M. persicae* consumed by one *H. axyridis* larva (±*SE*)			Development time (days ± *SE*)	Weights of newly emerged adults (mg ± *SE*)	
	2nd instar	3rd instar	4th instar		Female	Male
HAW	12.9 ± 0.9*	30.3 ± 1.8	67.2 ± 4.3	12.4 ± 0.1	22.2 ± 0.5*	17.5 ± 0.4*
HAM	9.7 ± 0.6	28.8 ± 1.6	65.1 ± 3.3	12.5 ± 0.2	20.1 ± 0.4	15.8 ± 0.4

*Note*

Values shown were mean ± *SE*, and asterisk indicates a significant difference between HAM and HAW (*p* < 0.05, *t* test).

#### 3.3.2 Reproductive capability

**Figure 4:**
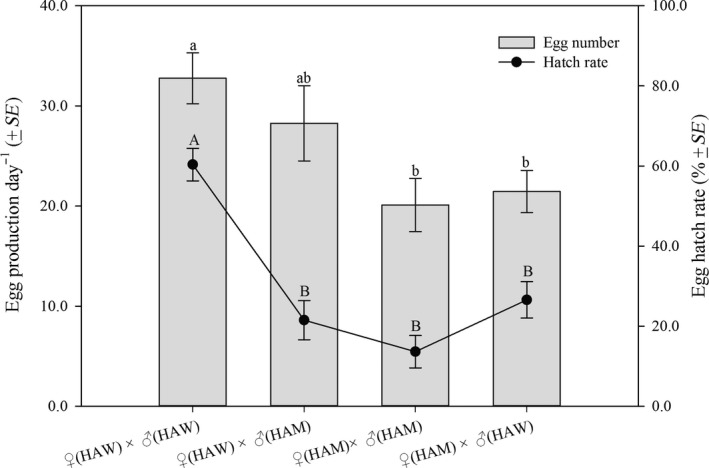
Reproductive capabilities of different crossing combinations of *Hamonia axyridis* mutant type (HAM) and wild type (HAW). Average number of egg production and hatch rate were recorded for 15 days from first oviposition. Different letters represent significant differences among the crosses (lowercase and uppercase letters were used for egg production and hatch rate, respectively) (*p *<* *0.05, Tukey's HSD test)

Significantly more eggs were produced by female HAW in the cross ♀(HAW) × ♂(HAW) (32.8) than those produced by HAM in the cross ♀(HAM) × ♂(HAM) (20.1) or ♀(HAM) × ♂(HAW) (21.4), but they all did not differ significantly from that produced by HAW in the cross ♀(HAW) × ♂(HAM) (28.3) (*F*_3, 47_ = 4.39, *p *=* *0.0087) (Figure 4). Similarly, the egg hatch rate in cross ♀(HAW) × ♂(HAW) (60.4%) was significantly higher than those in the cross ♀(HAM) × ♂(HAM) (13.6%), ♀(HAW) × ♂(HAM) (21.5%) or ♀(HAM) × ♂(HAW) (26.6%) (*F*_3, 47_ = 21.9, *p *<* *0.0001) (Figure 4).

#### 3.3.3 Drought and ultraviolet resistance

**Figure 5:**
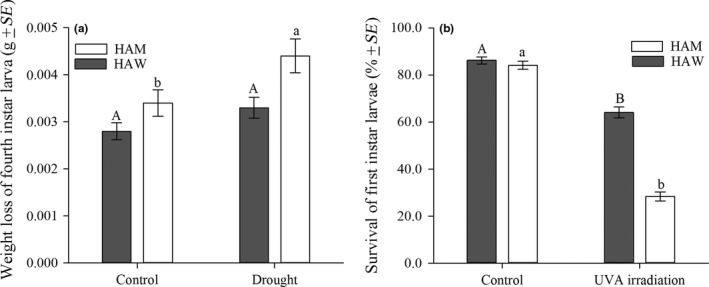
Resistance of *Harmonia axyridis* mutant (HAM) and wild type (HAW) to desiccation and ultraviolet (UVA) irradiation. (a) Weight loss of the fourth instar larvae after being restricted in extreme drought conditions for 36 hr; (b) Survival rate of 21st instar larvae after being exposed to UVA irradiation (365 nm) for 2 hr and then reared on *Myzus persicae* for 24 hr. Different letters represent significant differences between control and treatment (lowercase and uppercase letters were used for HAM and HAW, respectively) (*p *<* *0.05, Tukey's HSD test)

Desiccation was significantly affected by the coloration phenotype (*F*_1,99_ = 10.27, *p *=* *0.0018) and experimental condition (extreme drought or control) (*F*_1, 99_ = 7.86, *p *=* *0.0061). HAM had significantly more weight loss under drought conditions than under control conditions (*F*_1, 49_ = 4.83, *p *=* *0.0328), while such significant difference was not found for HAW (*F*_1, 49_ = 3.13, *p *=* *0.0832). Under control conditions, the weight loss was similar between HAM and HAW (*F*_1, 49_ = 3.38, *p *=* *0.0722), but HAM had significantly more weight loss after being exposed to the drought conditions (*F*_1, 49_ = 6.91, *p *=* *0.0115) (Figure 5a).

The survival of first instar larvae was significantly affected by body color (*F*_1, 99_ = 55.07, *p *<* *0.0001) and UV exposure (*F*_1, 99_ = 235.44, *p *<* *0.0001). For both HAM and HAW, the survival rates under UVA irradiation were significantly lower than those in control treatments (HAW: *F*_1, 49_ = 64.62, *p *<* *0.0001; HAM: *F*_1, 49_ = 464.85, *p *<* *0.0001). Compared to HAW, significantly fewer HAM survived after being exposed to UVA (*F*_1, 49_ = 141.80, *p *<* *0.0001), while such difference was not detected under the control condition (*F*_1, 49_ = 0.78, *p *=* *0.3822) (Figure 5b).

## 4 DISCUSSION

In this study, a series of crossing experiments confirmed that the abnormal phenotype *gr* was autosomal recessive (*grgr*). In addition, for the melanin synthesis precursors, the mutation HAM had a significantly lower concentration of L‐DOPA (*p *=* *0.0012), but had a higher concentration of dopamine compared to the wild‐type HAW (*p *=* *0.0235). Consequently, HAM larvae had light‐colored integument compared to HAW. Moreover, the abnormal phenotype was continuously maintained throughout development. This pattern is different from most melanization mutants which are generally expressed at one or two particular stages (Liu, Wang, & Li, 2015). In newly emerged adults, HAM individuals still had significantly lower concentration of L‐DOPA (*p *<* *0.0001), but no significant difference was detected for dopamine (*p *=* *0.9392). These results illustrated that *gr* was a special body color mutant deficient in melanin production, and the changes of L‐DOPA were consistent during early and fully developed stages. However, the changes of dopamine in HAM compared to HAW were different in 4th instar larvae and newly emerged adults, and the mechanism for this difference remains unknown.

The mutant HAM was inferior to HAW in resistance to both desiccation and UVA irradiation. Several studies have confirmed that UVA and desiccation resistance are related to melanin pigmentation intensity (Jacobs, 1985; Majerus, 1998; Matute & Harris, 2013). For example, *Drosophila* with greater melanization was reported to be more resistant to desiccation (Brisson, Toni, Duncan, & Templeton, 2005). In addition, the extent of melanism has been also reported to be positively associated with pathogen resistance (Wilson, Cotter, Reeson, & Pell, 2001). For *H. axyridis*, many species of entomopathogenic fungi could infect its larvae, pupae, and overwintering adults, but the susceptibility varies among life stages (Roy et al., 2011; Steenberg & Harding, 2009). Thus, the light‐colored mutant HAM might have weak pathogen resistance as compared to HAW, and different variations would be found on different life stages. On the other hand, in the absence of fungal infection, the heavy defense investments made by melanic morphs might result in trade‐offs of life‐history traits, such as biomass and fecundity (Dubovskiy et al., 2013; Wilson et al., 2001).

It was surprising to note that the biological characteristics of HAM were vastly different from those of HAW, and many parameters in HAM were comparatively inferior. Compared to HAW, relatively fewer *M. persicae* adults were consumed by the larvae of HAM, which resulted in the smaller body size of adults. For reproduction, both fecundity and egg hatch rate in the crosses ♀(HAM) × ♂(HAM) were significantly lower than those in ♀(HAW) × ♂(HAW) (*p *=* *0.0087 and *p *<* *0.0001, respectively), and the crosses between ♀(HAM) and ♂(HAM) had an extremely low egg hatch rate of 13.6%. In addition, mysterious interactions were found in the intercross of HAM and HAW. For example, the egg production and hatch rate of the crosses ♀(HAW) × ♂(HAM) were similar to those of ♀(HAM) × ♂(HAM) and ♀(HAM) × ♂(HAW), but were significantly less than those of ♀(HAW) × ♂(HAW). These differences might be caused by the following two reasons. First, cuticle melanism in insects has been reported to be linked to a number of life history traits, such as immune function, fecundity, and lifespan (Krams et al., 2016). Former studies showed that some melanin synthesis genes, for example, *tan*,* ebony,* and *yellow* as elucidated in *D. melanogaster*, are expressed not only in epidermal cells but also in neurons and neuron‐associated cells that could affect both body color and behavior (Beall & Hirsh, 1987; Drapeau, Radovic, Wittkopp, & Long, 2003; Wagner et al., 2007; Wilson, Goodman, & Strelets, 2008). Here, the feeding capability of HAM was affected and thus might influence body size and reproduction. On the other hand, melanin pigment is an important signal of quality, and variation in integument darkness might symbolize different quality (Niecke, Rothlaender, & Roulin, 2003). Meanwhile, reproductive investment in response to male quality is widespread in insects (Sheldon, 2000), and thus, HAM and HAW might have different reproductive investments in their intercrosses.

In addition, on the elytra of HAM, the dark spots were less melanization compared to HAW. Former studies showed that less melanization of elytra spots in *H. axyridis* (form *succinea*, the background of elytra is yellow or yellow with black spots) would be an adaption to high temperatures (Michie, Mallard, Majerus, & Jiggins, 2010), but it was uncertain whether there were any similar advantages for HAM. Here, the coloration of the orange background and the dark spots were simultaneously changed when compared to HAW. Specifically, the contents as well as total concentration of carotenoids in HAM greatly differed with those of HAW. Our results implied that the carotenoids‐ and melanin‐based elytra coloration should be highly correlated in *H. axyridis*, and this study might be the first to report the correlation of these two pigments in insects. Several studies in birds, which express both carotenoid‐ and melanin‐based plumage ornaments have found that both these two signals were influenced by oxidative stress (Alonso‐Alvarez & Galván, 2011; Henschen, Whittingham, & Dunn, 2016). In addition, for some insects, for example, aphids, the production of melanin, and carotenoid pigments were assumed to be controlled by juvenile hormone (Lees, 1966; Tsuchida, 2016).

In summary, this novel melanin‐deficient mutation was tightly associated with fitness changes. Our results further reveal that extensive pleiotropy can be caused by melanogenesis genes. More importantly, this research might be the first to report an interaction of melanin‐ and carotenoid‐based coloration in insects. This light‐colored mutant in *H. axyridis* might be a new model to study the molecular mechanism of melanism as well as the range and strength of pleiotropy.

## CONFLICT OF INTEREST

None declared.

## AUTHOR CONTRIBUTIONS

Y.X.S. and T.X.L. designed research; Y.X.S. and Y.N.H. performed research; Y.Y. and Y.Z. provided assistance; and Y.X.S., Y.N.H., Y.F., and T.X.L. wrote the manuscript.

## DATA ACCESSIBILITY

Data available from the Dryad Digital Repository: https://doi.org/10.5061/dryad.04b3g81
