# Supplementary figures and images for: Morphological and biological characterization of a light‐colored mutant in the multicolored Asian lady beetle, Harmonia axyridis
Source: Ecol Evol. 2018 Oct 3;8(20):9975–85. doi: 10.1002/ece3.4379 (PMC6206217; doi:10.1002/ece3.4379)

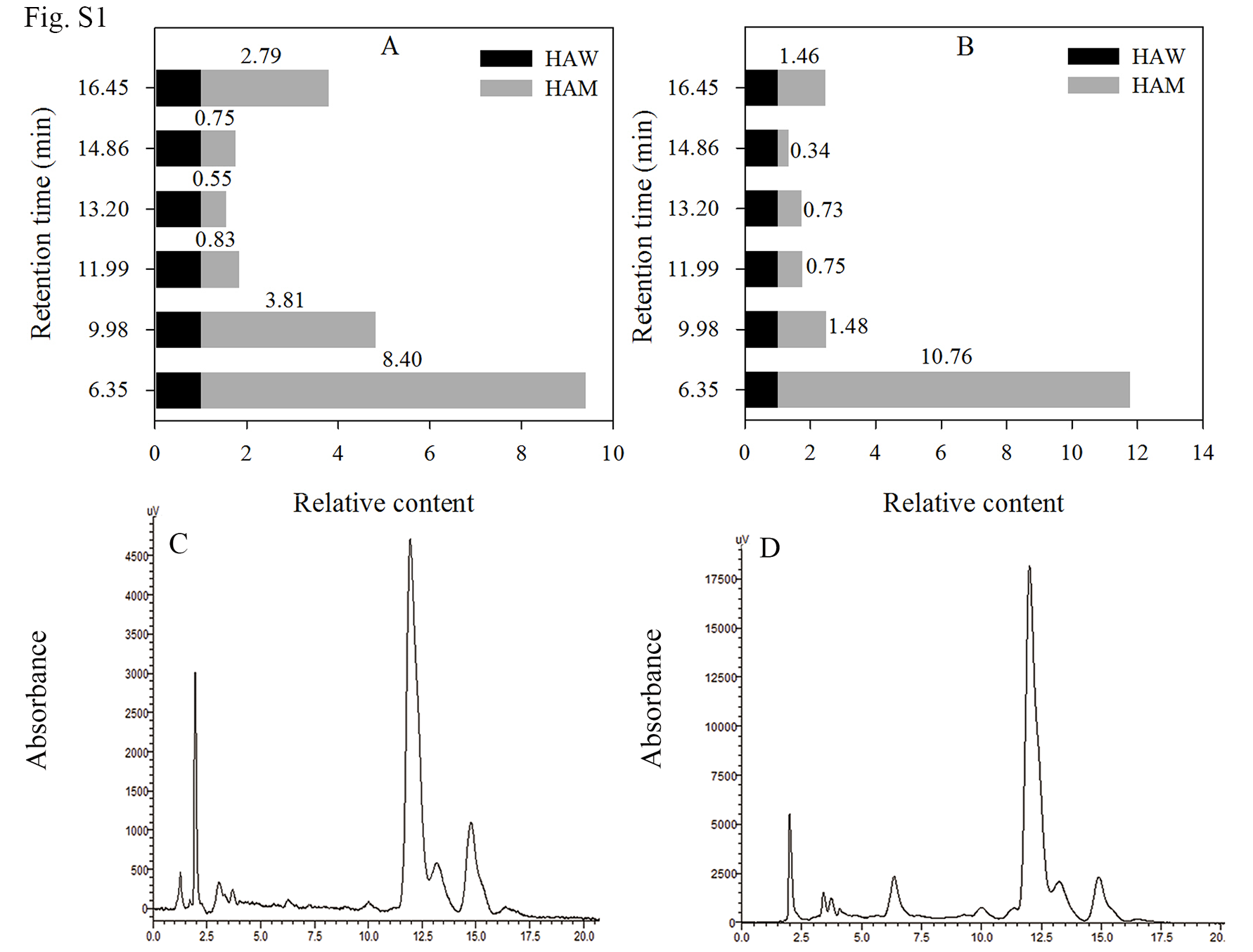

Supplement: Supplementary file 1 [file ECE3-8-9975-s001.tif]
