# Supplementary material for: Morphological and biological characterization of a light‐colored mutant in the multicolored Asian lady beetle, Harmonia axyridis
Source: Ecol Evol. 2018 Oct 3;8(20):9975–85. doi: 10.1002/ece3.4379 (PMC6206217; doi:10.1002/ece3.4379)
Supplement: Supplementary file 2 [file ECE3-8-9975-s002.doc]

**Supplemental information**

Materials and methods

*Main components of carotenoids in the elytra*

Five to six right elytra were weighted (7 to 9 mg) and extracted with the method as described in section *The phenotype* *and physiological changes*. The dry residues were re-dissolved in 0.5 ml of the initial mobile phases (30% acetonitrile + 70% methyl alcohol (v:v)). The solution was passed through a 0.22 μm filter before injection for analysis. Carotenoids in the elytra were analyzed through a high-performance liquid chromatography (HPLC) (SHIMADZU LC-2010A, Japan) fitted with Wondasil C18 Column (125 Å pore size, 5 mm, 150 mm × 4.6 mm; SHIMADZU, Japan), and the detection wavelength was 450 nm. Carotenoids elution was performed applying a six-step gradient at a flow rate of 1 ml min^-1^ (Table S1). The mobile phase A, B was methyl alcohol and acetonitrile (chromatographic grade, Riedel de Haen, Seelze, Germany), respectively. Three certified analytical standards α-carotene (> 99.99%, Sigma-Aldrich Chemical Co (St. Louis, MO)), β-carotene (> 98%, Yuanye Bio-Technology Co., Ltd (Shanghai, China)) and lycopene (> 90%, Yuanye Bio-Technology Co., Ltd (Shanghai, China)) were used to identify the components of carotenoids in the elytra.

Following the chromatogram of the three mixing standards, we found that the retention time of lycopene, α-carotene and β-carotene was at around 9.98 min, 13.80 min and 14.85 min, respectively. However, in this study, several other kinds of carotenoids could not be identified, and the relative content ((peak area/weight of the elytra) /(peak area/weight of the elytra of HAW)) of each carotenoid species was used to make a comparison between HAM and HAW.

Table S1 The program of grade elution of HPLC

| Time (min) | Mobile phase A  (percent of volume) | Mobile phase B  (percent of volume) |
| --- | --- | --- |
| 0 | 70% | 30% |
| 5 | 70% | 30% |
| 20 | 20% | 80% |
| 25 | 20% | 80% |
| 26 | 70% | 30% |
| 35 | 70% | 30% |

Figure legend

Figure S1 The content of carotenoids in the elytra of *Harmonia axyridis* mutant type (HAM) and wild type (HAW). Relative content of carotenoids in the elytra of HAM was calculated following the equation: Relative content of carotenoids = (peak area/weight of the elytra) /(peak area/weight of the elytra of HAW). Here, the content of carotenoid in the elytra of HAW was defined as 1. A: females; B: males; C and D were the HPLC chromatogram with a C18 column of the carotenoids extracted from the elytra of female HAW and HAM, respectively.
